# Evaluating the Visible Difference Parenting Toolkit: an ACT-based intervention to improve parental/carer stress and psychosocial well-being of carers of children with appearance affecting conditions and injuries

**DOI:** 10.1093/jpepsy/jsag043

**Published:** 2026-07-11

**Authors:** Maia Thornton, Emma Waite, Paul White, Anna Zarola, Nymfodora Antoniou, Alex Clarke, Diana Harcourt

**Affiliations:** Centre for Appearance Research, School of Social Sciences, College of Health, Science & Society, University of the West of England, Bristol, United Kingdom; Centre for Appearance Research, School of Social Sciences, College of Health, Science & Society, University of the West of England, Bristol, United Kingdom; Centre for Appearance Research, School of Social Sciences, College of Health, Science & Society, University of the West of England, Bristol, United Kingdom; Centre for Appearance Research, School of Social Sciences, College of Health, Science & Society, University of the West of England, Bristol, United Kingdom; Centre for Appearance Research, School of Social Sciences, College of Health, Science & Society, University of the West of England, Bristol, United Kingdom; Centre for Appearance Research, School of Social Sciences, College of Health, Science & Society, University of the West of England, Bristol, United Kingdom; Centre for Appearance Research, School of Social Sciences, College of Health, Science & Society, University of the West of England, Bristol, United Kingdom

**Keywords:** psychosocial intervention, parent psychosocial functioning, coping skills and adjustment, eHealth/mHealth/digital health, evidence-based practice, visible difference

## Abstract

**Objective:**

Alongside the typical challenges of parenting, parents and carers of children with visible differences can experience additional psychosocial challenges including anxiety, stress, guilt, and concerns about stigma and discrimination. The Visible Difference Parenting Toolkit is an ACT-based intervention aimed at improving psychosocial well-being for this group of parents/carers. This study examined the acceptability and potential benefit of the intervention utilizing a mixed-methods single-arm pre-post design.

**Method:**

Parents/carers (baseline *n *= 173) completed an online survey at three time points: (1) baseline, (2) post-intervention, and (3) 2-month follow-up. Paired *t*-tests on available matched data assessed differences in outcome measures between timepoints (83 pairs at post-intervention, 62 at follow-up). Open-ended responses were analyzed using content analysis.

**Results:**

Statistically significant reductions in parent-reported depression and anxiety and statistically significant improvements in parent-reported self-efficacy were found between baseline and post-intervention. Additionally, significant reductions in parent-reported depression and anxiety and significant improvements in self-compassion, self-efficacy, and psychological flexibility were found between baseline and follow-up. Parenting stress did not significantly improve post-intervention immediately, but showed significant change at follow-up. Responses to open-ended questions indicated that although some ongoing concerns remained about future challenges, the intervention helped parents/carers feel more knowledgeable and confident in supporting their child.

**Conclusions:**

This study suggests the Visible Difference Parenting Toolkit is an acceptable and potentially useful intervention for supporting parents/carers of children with a visible difference. This novel intervention addresses a gap in the current support provision in the visible difference field and is now freely available through www.VisibleDifferenceSupportHub.com.

A visible difference is defined as an appearance that differs from the “norm” (Rumsey & Harcourt, 2004) and can be caused by a wide range of health conditions (e.g., congenital conditions such as cleft lip and/or palate; skin conditions such as eczema and vitiligo), acquired injuries (e.g., burns and limb loss), or medical treatments (e.g., surgical scarring and chemotherapy-related hair loss). Regardless of its cause or nature, the psychosocial impact of living with a visible difference can be significant, including negative self-perceptions, poor body image, low self-esteem, fear of negative evaluation, and anxiety (Jenkinson et al., 2015; Waite et al., 2024). Parents and carers of children and young people with visible differences can also face psychosocial challenges as a consequence of the child’s condition or injury (Thornton et al., 2021), including shock, stress, anxiety, depression, or guilt (Hawkins et al., 2019; Heath et al., 2018; Stock & Billaud Feragen, 2019). Parents/carers can feel anxious about actual or anticipated social stigma, such as unwanted attention (e.g., staring, comments, and questions), teasing or bullying (Ablett & Thompson, 2016; Hlongwa & Rispel, 2018; Oliver et al., 2020), as well as concerns about communicating with their child about their visible difference (Zelihić et al., 2021), practical challenges such as attending hospital appointments (Billaud Feragen et al., 2022) and the stress of ongoing medical treatment and decision-making (Heath et al., 2018). Further, low confidence in parenting skills (i.e., parenting self-efficacy) in parents/carers of children with chronic health conditions is associated with psychological distress and health-related quality of life measures, including fatigue (Giallo et al., 2013; Harper et al., 2013). Consequently, there is a need for psychosocial support for parents/carers of children with appearance-affecting conditions and injuries.

Although some interventions for parents/carers of children with appearance-affecting conditions and injuries exist, many focus on skills training for symptom management (e.g., the Triple P Parenting Program; Morawska et al., 2016) and do not provide psychosocial support for the parents/carers themselves. A systematic review of interventions aimed at improving psychosocial outcomes for parents/carers of children with skin conditions found moderate to strong evidence for the effectiveness of 10 interventions (e.g., Fordyce Happiness Program, Triple P Parenting Program, Early Family Intervention Program; Costa et al., 2021), though methodological quality varied, with several studies assessed as weak. As such, there remains a support gap for this population.

There is a growing body of evidence for acceptance and commitment therapy (ACT) as an appropriate therapeutic approach for people whose lives are affected by visible differences (e.g., Thornton et al., 2026; Zucchelli et al., 2018, 2021), as well as parents/carers of children with long-term health conditions (e.g., Jin et al., 2021). ACT is a third-wave cognitive-behavioral approach that encourages individuals to build awareness of personal values and commit to living in accordance with them (Hayes et al., 2006). It teaches strategies to manage difficult thoughts and feelings that may arise and prevent individuals from taking valued action. ACT targets six core psychological processes (acceptance, cognitive defusion, being present, self-as-context, values, and committed action) with the overall aim of fostering psychological flexibility (the ability to orient to the present moment with openness and awareness and behave in accordance with one’s values; Hayes et al., 2006).

The Visible Difference Parenting Toolkit, detailed below, is a self-guided e-book based on ACT concepts. An acceptability study, informed by the Theoretical Framework of Acceptability (Sekhon et al., 2018), included the eHealth Impact Questionnaire (Kelly et al., 2015), the National Health Service (NHS) Friends and Family Test (NHS, 2012), and user engagement questions including time spent reviewing the materials, which sections participants had engaged with, and which they had found most useful. Data from 22 parents/carers of children with a range of visible differences demonstrated that both the content and the format of the toolkit were very acceptable, and parents/carers reported feeling confident using the intervention and said they would be either “Likely” or “Very Likely” to recommend it to others (see Thornton et al., 2026, for full details of this acceptability study). In line with Medical Research Council guidance for intervention development (Skivington et al., 2021), it was important to establish the potential usefulness of this new intervention.

The present study aimed to assess the potential benefit of the Visible Difference Parenting Toolkit (an ACT-based e-book intervention for parents and carers of children with a visible difference), using a mixed-methods survey with data collection at three time points. The primary research question was, “Is the intervention effective at reducing stress in parents and carers of children with a visible difference?” It was hypothesized that:

1. There will be a decrease in parent-reported stress, anxiety, and depression between baseline and post-intervention and between baseline and follow-up.
2. There will be an increase in parent-reported psychological flexibility, self-compassion, and self-efficacy between baseline and post-intervention and between baseline and follow-up.

## Materials and Methods

### Study design

This study utilized a concurrent mixed-methods approach (Creswell, 2021) to evaluate the potential benefit of an evidence-based intervention for parents and carers of children with a visible difference of any kind. Quantitative data were collected via online questionnaires at three time points: (1) baseline, (2) post-intervention, and (3) 2-month follow-up. Qualitative data were collected from open-ended survey questions and a sub-sample via one-to-one online interviews with the first author. The questionnaires included standardized measures of psychosocial outcomes and user engagement. Open-ended questions allowed participants to elaborate on their experiences in their own words (Greene et al., 1989). A completed CONSORT checklist is available in the Supplementary material.

### Participants

Any adult with caring or guardianship responsibilities for a child under 18 with an appearance-affecting condition or injury was eligible. Member organizations of the Appearance Collective, a group of UK-based charities supporting individuals and families affected by visible difference, were approached to assist with recruitment. These organizations shared a research advert drafted by the researchers via social media, websites, newsletters, and events. Research adverts were also sent to members of the Centre for Appearance Research participant pool and social media pages. The adverts included information about the aim of the study, who was eligible, what participation entailed (e.g., receiving the toolkit, taking part in a series of surveys), and instructions for accessing further information and the baseline survey. Steps were taken to help prevent imposter participants (see Schneider et al., 2025). Specifically, organizations were asked only to share the research advert with closed social media groups, rather than public pages. Additionally, open-ended responses and answers to demographic questions were closely monitored to help identify and remove any imposter participants who provided nonsensical responses.

### Patient and public involvement

An advisory group of two parents with lived experience of caring for a child with an appearance-affecting condition or injury was recruited via the Appearance Collective (see above). The advisory group provided feedback through online one-to-one meetings at three time points: during the initial development of the research questions and study design and when deciding on outcome measures; during preparation to review the study materials; and during the study for guidance on recruitment methods to increase participation and minimize attrition. A summary of the findings was shared with the group, and members were compensated for their time.

### Intervention materials

The Visible Difference Parenting Toolkit is a self-guided e-book developed with parents/carers with lived experience, following a participatory action research approach (see Thornton et al., 2026 for details of the intervention development). The 60-page e-book, currently only available in English, includes information, exercises, and links to audio/video content. Content was informed by existing evidence and theory (e.g., Billaud Feragen et al., 2022; Heath et al., 2018; Thornton et al., 2021, 2024; Zelihić et al., 2021) and divided into two sections: (1) ACT principles and “helper skills” for managing difficult thoughts and feelings; and (2) guidance and exercises related to key constructs in parenting and visible difference research (e.g., social challenges, communication, and support). The six ACT processes informing the intervention were as follows: (1) experiential acceptance; (2) cognitive defusion (ability to detach from or not ‘fuse’ with thoughts, images, memories); (3) being present (ability to consciously and flexibly attend to what is happening in the present); (4) values (desired qualities or psychological action); (5) committed action (behavior in accordance with an individual’s values); and (6) self-as-context (observing cognitions, emotions, behavior; Harris, 2006). The textual intervention content has been assessed to be presented at a reading age of 12 years old or 8th grade.

### Outcome measures

#### Psychosocial outcomes

*Anxiety and depression:* The Hospital Anxiety and Depression Scale (HADS; Zigmond & Snaith, 1983) is a 14-item scale with two subscales: anxiety and depression. Items are scored from 0 (*not at all*) to 3 (*most of the time*) in response to the past week, with higher scores indicating greater distress. A score of 11 or higher indicates clinical levels of anxiety or depression. Internal consistency was acceptable to good across baseline, post-intervention, and follow-up: depression (α = .82, .68, .80) and anxiety (α = .83, .76, .85).

*Parent stress:* The Paediatric Inventory for Parents Short Form (PIP-SF; Larson et al., 2020) is a measure of parenting stress related to caring for a child with a chronic illness over the previous 7 days. It includes 13 items across four subscales: communication, medical care, role function, and emotional distress. Items are scored from 1 (*not at all*) to 5 (*extremely*), with higher scores indicating greater stress. Internal consistency was good across baseline (α = .85), post-intervention (α = .89), and follow-up (α = .91).

*Parent self-efficacy:* A previous study investigating risk and protective factors of parents/carers of children with an appearance-affecting condition or injuries (Thornton et al., 2024) constructed an 11-item self-efficacy scale specific to the needs and experiences of parents of children with visible differences. Items are based on concerns highlighted in existing qualitative research (e.g., teasing, parent–child communication; Thornton et al., 2021). Example items include: “I can support my child in telling others about their condition,” “I can promote confidence and resilience,” and “I can support treatment decisions.” Items are scored from 1 (*not at all confident*) to 10 (*very confident*), with higher scores indicating greater self-efficacy. Internal consistency was strong across baseline (α = .87), post-intervention (α = .91), and follow-up (α = .93).

*Self-compassion:* The Self-Compassion Scale Short-Form (SCS-SF; Raes et al., 2011) is a 12-item scale scored from 1 (*almost never*) to 5 (*almost always*), with higher scores indicating greater self-compassion. Internal consistency was good across baseline (α = .87), post-intervention (α = .84), and follow-up (α = .79).

*Psychological flexibility:* The Comprehensive assessment of ACT processes (CompACT; Francis et al., 2016) is a 23-item general measure of psychological flexibility as conceptualized within the ACT model, with three subscales: openness to experience, behavioral awareness, and valued action. Items are scored from 0 (*strongly disagree*) to 6 (*strongly agree*), with higher scores indicating greater flexibility. Internal consistency was good across baseline, post-intervention, and follow-up: full scale (α = .88, .84, .87); openness to experience (α = .82, .76, .71); awareness (α = .83, .81, .78); and valued action (α = .84, .80, .85).

*Valued action:* The Valued Living Questionnaire (VLQ; Wilson & Groom, 2006) was included to capture valued action specific to parenting. It is a 20-item scale that asks respondents to rate 10 different life domains, indicating their level of importance and how consistently they have lived in accordance with these values in the last week. Items are scored on a 10-point scale ranging from 1 (*not at all important/not consistent with my value*) to 10 (*extremely important/completely consistent with my value*). Three domains from the scale were included, asking participants to reflect on “Parenting,” “Marriage/couples/intimate relationships,” and “Family (other than marriage or parenting)”.

#### User engagement measures

The post-intervention survey included four questions about engagement. Participants were asked which toolkit sections they used, whether they completed any exercises, and which ones. They were also invited to suggest alternative formats for the toolkit.

#### Open-ended questions

Open-ended questions were included at the end of the post-intervention and follow-up surveys. They asked, “Do you have anything else that you’d like to share?” to allow participants to elaborate and provide deeper context (O’Cathain & Thomas, 2004).

### Sample size

A pre-study effect size consistent with study hypotheses was conservatively assumed to be in the small-to-medium range (Cohen’s *d *= 0.35–0.40). We anticipated that approximately one third of eligible and consenting participants would not provide post-intervention data, with a further one-in-four of the post-intervention completers failing to provide follow-up data. On this basis, we aimed to recruit at least 150 participants to achieve approximately 100 post-intervention completers and 75 follow-up completers. These sample sizes would provide >90% power to detect an effect of *d *= 0.40 and approximately 85–90% power for *d *= 0.35 (two-sided paired *t*-tests, α = .05).

### Procedure

**Figure 1:**
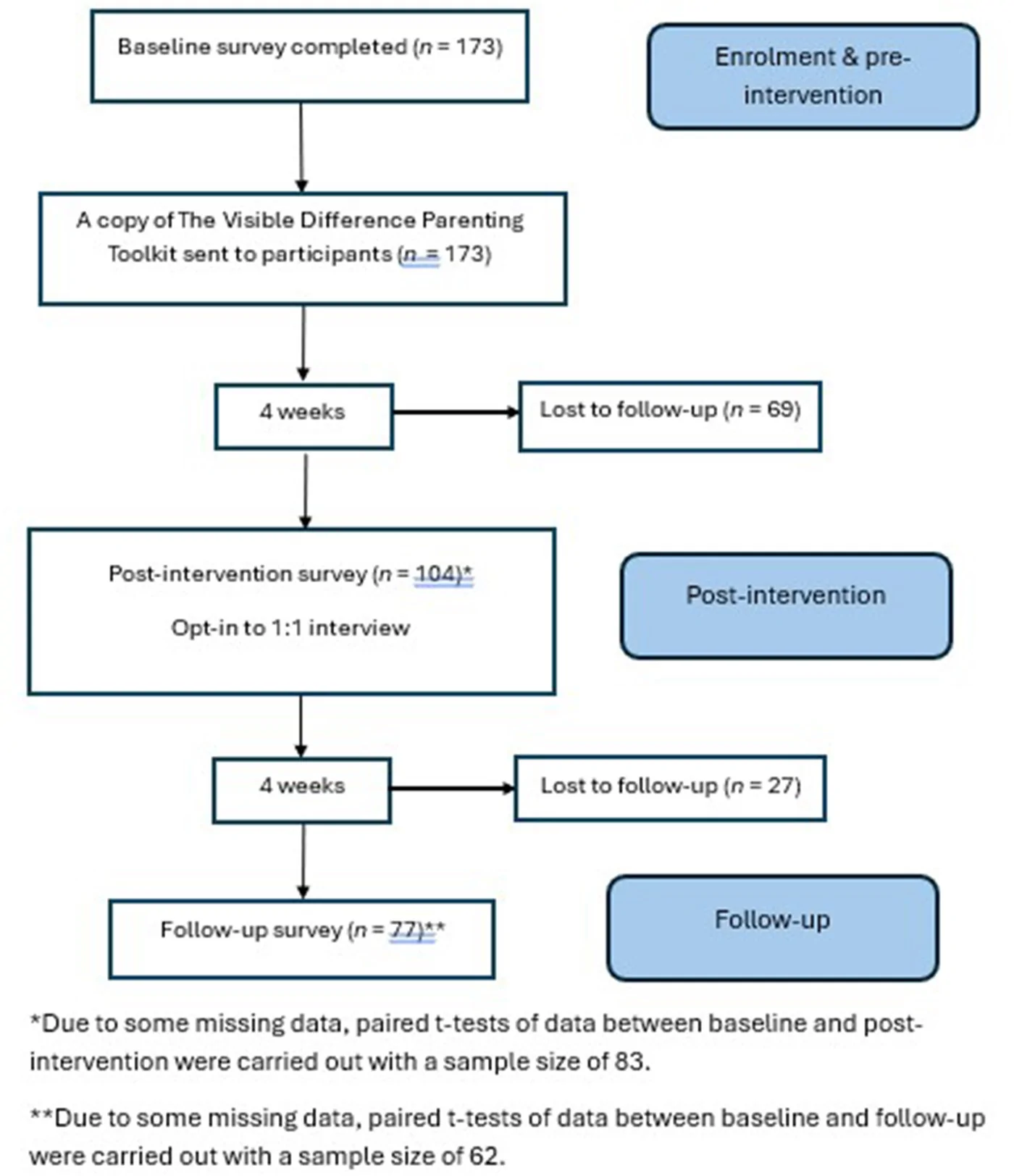
Flow diagram of study procedure and participant numbers.

This research project received ethical approval from the University of the West of England College of Health, Science and Society Research Ethics Committee (ref no: HAS.23.03.087). Eligible parents and carers were given a detailed information sheet outlining the content and format of “The Visible Difference Parenting Toolkit” and the evaluation procedure. After reviewing the information sheet, those wishing to participate provided written informed consent via an online form. Participants completed baseline measures hosted on a Qualtrics survey and were then emailed a PDF copy of the intervention. The toolkit is a self-guided intervention, and parents were instructed to read the content and practice the exercises independently. While there was no set timeframe or suggested pacing for completing this, participants were informed that the research team would be back in touch in “a few weeks” to find out how things had gone, implying that they should have completed at least some of the intervention by then. Four weeks later, participants were emailed a link to complete post-intervention outcome measures, also hosted on Qualtrics. At the end of the survey, they were asked if they wished to opt in for a one-to-one interview with a researcher about their experience of using The Visible Difference Parenting Toolkit. All those who opted in and provided consent were interviewed (data presented in another publication). Two months after baseline, participants were sent a link to complete the follow-up outcome measures. After completing the post-intervention and follow-up surveys, participants received a £10 shopping voucher as a thank you (total £20). Those who took part in an interview received an additional £20 voucher. Figure 1 summarizes participant flow through the study.

### Data analysis

#### Outcome measures

The study design (single group pre-, post-, and follow-up) was analyzed using established statistical techniques. Paired-samples *t*-tests assessed changes in mean response between baseline and post-intervention and baseline and follow-up. Standardized effect sizes for repeated measures were used to estimate Cohen’s *d*, using formula 10 in Lakens (2013), where *d* values are interpreted as very small (0.01–0.2), small (0.2–0.5), medium (0.5–0.8), large (0.8–1.2), and very large (>1.2; Sawilowsky, 2009). Paired analyses (McNemar test) compared the percentage of participants at baseline, post-intervention, and follow-up scoring 11 or higher on the HADS (an indication of levels of clinical anxiety or depression). Correlations were carried out between changes in psychological flexibility and changes in depression and stress from baseline to post-intervention and from baseline to follow-up. Unfortunately, we experienced some technical difficulties in matching baseline data with post-intervention and follow-up data. This prevented paired t-tests from being conducted for these participants, since we only proceeded to only use paired data where we were confident in the matching. This resulted in sample sizes of 83 and 62 for the analyses at post-intervention and follow-up, respectively.

A missing values analysis was conducted. Participants providing no post-intervention data were considered dropouts and compared to those who did, using χ^2^ tests for categorical demographics and independent samples *t*-tests for baseline scale data. No significant differences were found. Multiple imputed chained equations (100 imputations) were used to address missing data, with sensitivity analyses reported as Supplementary material. User engagement data are presented descriptively. All analyses were conducted using SPSS version 29. Two-sided tests were used throughout with a nominal significance level of α = .05.

#### Open-ended responses

Open-ended survey responses were analyzed using content analysis. The fourth and fifth authors extracted data from Qualtrics and inputted it into two Microsoft Word tables: one for post-intervention data and one for follow-up. They familiarized themselves with the data through repeated readings before generating initial codes. These codes were refined through further readings of the data set. Halfway through coding, the authors compared notes to ensure consistency. After coding the full dataset, they discussed and justified their codes, then generated candidate categories and sub-categories. These were reviewed with the first author before finalizing.

## Results

In total, 173 parents and carers were recruited at baseline. Of these, 126 were women, 24 were men, 1 was non-binary, and 22 did not report gender. The mean participant age was 38.8 years (range = 18–56, *SD *= 6.73). Eight participants identified as Asian/Asian British, eight as Black/African/Caribbean/Black British, eight as mixed/multiple ethnic groups, and 123 as White. The mean age of their child with a visible difference was 8.5 years (range = 1–18, *SD *= 4.79), with conditions including birthmarks, skin conditions, craniofacial differences, burn injuries, and hair loss. See Table 1 for demographic details. Twenty parents/carers (19 women, 1 non-binary person; 18 White, one White and Asian, 1 Black), aged 29–53 years (*M *= 37.0, *SD *= 6.78), also provided 29 responses to open-ended survey questions: 16 at post-intervention and 13 at follow-up.

**Table 1:** Summary of participant demographics.

		*N*
Gender	Female	126
	Male	24
	Non-binary	1
	Missing	22
Age	18–56 (*M *= 38.83, *SD *= 6.73)	150
	Missing	23
Ethnicity	Asian/Asian British	8
	Black/African/Caribbean/Black British	8
	Mixed/Multiple ethnic groups	8
	White	123
	Other ethnic group	4
	Missing	22
Country	Australia	2
	Belgium	1
	Canada	2
	Ireland	1
	New Zealand	2
	Qatar	1
	Serbia	1
	UK	117
	USA	4
	Missing	41
Child’s visible difference	Albinism	1
	Birthmark	15
	Burn injury	16
	Craniofacial condition	15
	Ectodermal dysplasia	6
	Facial difference	2
	Facial paralysis	12
	Hair loss	23
	Jacobsen syndrome	1
	Microphthalmia, anophthalmia, and coloboma	3
	Neurofibromatosis	6
	Scarring	1
	Skin condition	48
	Spinal injury	2
	Williams syndrome	1
	Missing	22
Child’s age	1–18 (*M *= 8.50, *SD *= 4.79)	123
	Missing	50

Missing values analysis indicated that those consenting to the study but not providing post-intervention outcome data did not significantly differ from those retained, either on demographic characteristics or baseline well-being measures. These dropouts were consistent with missing completely at random. Therefore, statistical comparisons are based on available case data, with corresponding comparisons on a multiple imputed data set provided in the Supplementary material, noting that the same broad conclusions are obtained under both analysis data sets. Table 2 summarizes the sample size and the mean and standard deviation for each measure at baseline, post-intervention, and follow-up. Table 3 presents the paired-samples *t*-test analyses and estimated effect sizes. Although 104 participants completed the post-intervention survey and 77 completed the follow-up survey, paired inferential analyses were restricted to 83 and 62 matched cases, respectively. Summary statistics for the imputed data are provided in Supplementary Table S1.

**Table 2:** Means and standard deviations for outcome variables.

Measure	Baseline			Post-intervention			Follow-up		
	*N*	Mean	*SD*	*N*	Mean	*SD*	*N*	Mean	*SD*
Depression	151	8.23	4.24	104	7.64	3.60	77	7.12	4.06
Anxiety	151	9.87	4.19	104	9.27	3.91	77	8.95	4.63
Parenting stress	151	39.44	9.41	103	38.58	10.58	77	37.22	11.44
Self-compassion	151	2.73	0.75	100	2.93	0.71	77	3.10	0.76
Psychological flexibility	151	73.89	16.98	102	74.84	15.27	77	78.26	16.16
Valued living	151	65.76	19.09	100	64.99	18.86	77	67.49	18.00
Self-efficacy	151	78.74	20.17	100	84.62	15.51	77	87.73	13.33
**Psych-flex subscales**									
Openness to experience	151	28.86	9.18	102	28.62	8.37	77	30.27	7.74
Behavioral awareness	151	14.85	5.46	102	14.54	5.44	77	15.03	5.22
Valued action	151	30.17	7.07	102	31.69	6.59	77	32.96	6.88

**Table 3:** Summary of paired *t*-tests comparing baseline to post-intervention and baseline to follow-up.

Baseline to post-intervention						
Measure	Baseline	Post-intervention	*t*	*df*	*p*	*d*
	Mean (*SD*)	Mean (*SD*)				
Depression	8.45 (4.45)	7.30 (3.68)	2.43	82	.017	0.28
Anxiety	9.99 (4.19)	8.64 (3.81)	3.11	82	.003	0.34
Parenting stress	39.39 (9.83)	37.24 (11.02)	1.78	81	.078	0.21
Self-compassion	2.84 (0.77)	2.97 (0.77)	−1.92	78	.059	0.17
Psychological flexibility	74.43 (17.86)	76.35 (16.48)	−1.22	80	.224	0.11
Valued living	66.39 (19.14)	68.43 (19.13)	−0.96	78	.341	0.11
Self-efficacy	80.94 (20.86)	86.48 (16.31)	−2.90	78	.005	0.30
**Psych-flex subscales**						
Openness to experience	28.64 (10.12)	29.40 (8.90)	−0.91	80	.367	0.08
Behavioral awareness	14.90 (5.66)	15.10 (5.79)	−0.34	80	.739	0.03
Valued action	30.89 (6.71)	31.85 (6.93)	−1.33	80	.187	0.14

Baseline to follow-up						
	Baseline Mean (*SD*)	Follow-up Mean (*SD*)	*t*	*df*	*p*	*d*
Depression	8.21 (4.29)	6.63 (4.26)	2.82	61	.007	0.37
Anxiety	10.01 (4.20)	7.89 (4.47)	3.71	61	<.001	0.49
Parenting stress	39.42 (10.08)	34.81 (11.45)	3.13	61	.003	0.43
Self-compassion	2.78 (0.81)	3.12 (0.84)	−3.52	61	<.001	0.41
Psychological flexibility	74.69 (17.81)	80.21 (17.26)	−2.42	61	.018	0.31
Valued living	67.05 (19.64)	71.46 (17.01)	−1.81	61	.076	0.24
Self-efficacy	82.02 (19.86)	89.85 (13.56)	−3.33	61	.001	0.47
**Psych-flex subscales**						
Openness to experience	27.98 (10.02)	31.02 (8.22)	−2.68	61	.009	0.33
Behavioral awareness	15.60 (5.87)	15.84 (5.28)	−0.37	61	.715	0.04
Valued action	31.11 (6.74)	33.35 (7.37)	−2.50	61	.015	0.32

*Note*. Some data were missing for participants due to a technical issue, resulting in 21 participants for whom data were available at post-intervention but not baseline, and 15 for whom data were available at follow-up but not baseline or post-intervention. This prevented paired *t*-tests from being conducted for these participants, resulting in sample sizes of 83 and 62 for the above analyses.

### Baseline and post-intervention

Paired-samples *t*-tests showed statistically significant decreases in depression, *t(*82) = 2.43, *p* = .017, *d *= 0.28, and anxiety, *t*(82) = 3.11, *p* = .003, *d *= 0.34, and a statistically significant increase in self-efficacy, *t*(78) = −2.90, *p* = .005, *d *= 0.30. No significant differences were found for parenting stress, self-compassion, psychological flexibility, or valued living. See Table 3 for a summary of the analysis.

### Baseline and follow-up

Between baseline and follow-up, statistically significant decreases were found in depression, *t*(61) = 2.82, *p* = .007, *d *= 0.37; anxiety, *t*(61) = 3.71, *p* < .001, *d *= 0.49; and parenting stress, *t*(61) = 3.13, *p* = .003, *d *= 0.43. Significant increases were observed in self-efficacy, *t*(61) = −3.33, *p* = .001, *d *= 0.47; self-compassion, *t*(61) = −3.52, *p* < .001, *d *= 0.41; openness to experience, *t*(61) = −2.68, *p* = .009, *d *= 0.33; and valued action, *t*(61) = −2.50, *p* = .015, *d *= 0.32, but not behavioral awareness, *t*(61) = −0.37, *p* = .715, *d *= 0.04. No significant differences were found for valued living. See Table 3 for details.

The same pattern of statistical conclusions was obtained in the imputed data set and is provided in Supplementary Table S2.

High levels of clinical anxiety were observed at baseline, where approximately 44% scored 11 or higher on the HADS anxiety subscale. In paired analyses, the corresponding percentage post-intervention was 29% (*p* = .015) and 35% at follow-up (*p* = .383). High levels of clinical depression were observed at baseline. Approximately 35% scored 11 or higher on the HADS depression subscale. In paired analyses, the corresponding percentage post-intervention was 21% (*p* = .007) and 18% at follow-up (*p* = .013).

### Correlation with changes in psychological flexibility

Changes in psychological flexibility from baseline to post-intervention were significantly correlated with corresponding changes in depression (*r *= −.373, *p* < .001), anxiety (*r *= −.465, *p* < .001), parenting stress (*r *= −.420, *p* < .001), self-compassion (*r* = .685, *p* < .001), parenting self-efficacy (*r* = .318, *p* = .004), and valued action (*r* = .275, *p* = .014).

Changes in psychological flexibility between baseline and follow-up were significantly correlated with corresponding changes in depression (*r* = −.531, *p* < .001), anxiety (*r* = −.579, *p* < .001), parenting stress (*r *= −.573, *p* < .001), self-compassion (*r* = .703, *p* < .001), parenting self-efficacy (*r* = .691, *p* < .001), and valued action (*r* = .374, *p* = .003).

**Figure 2:**
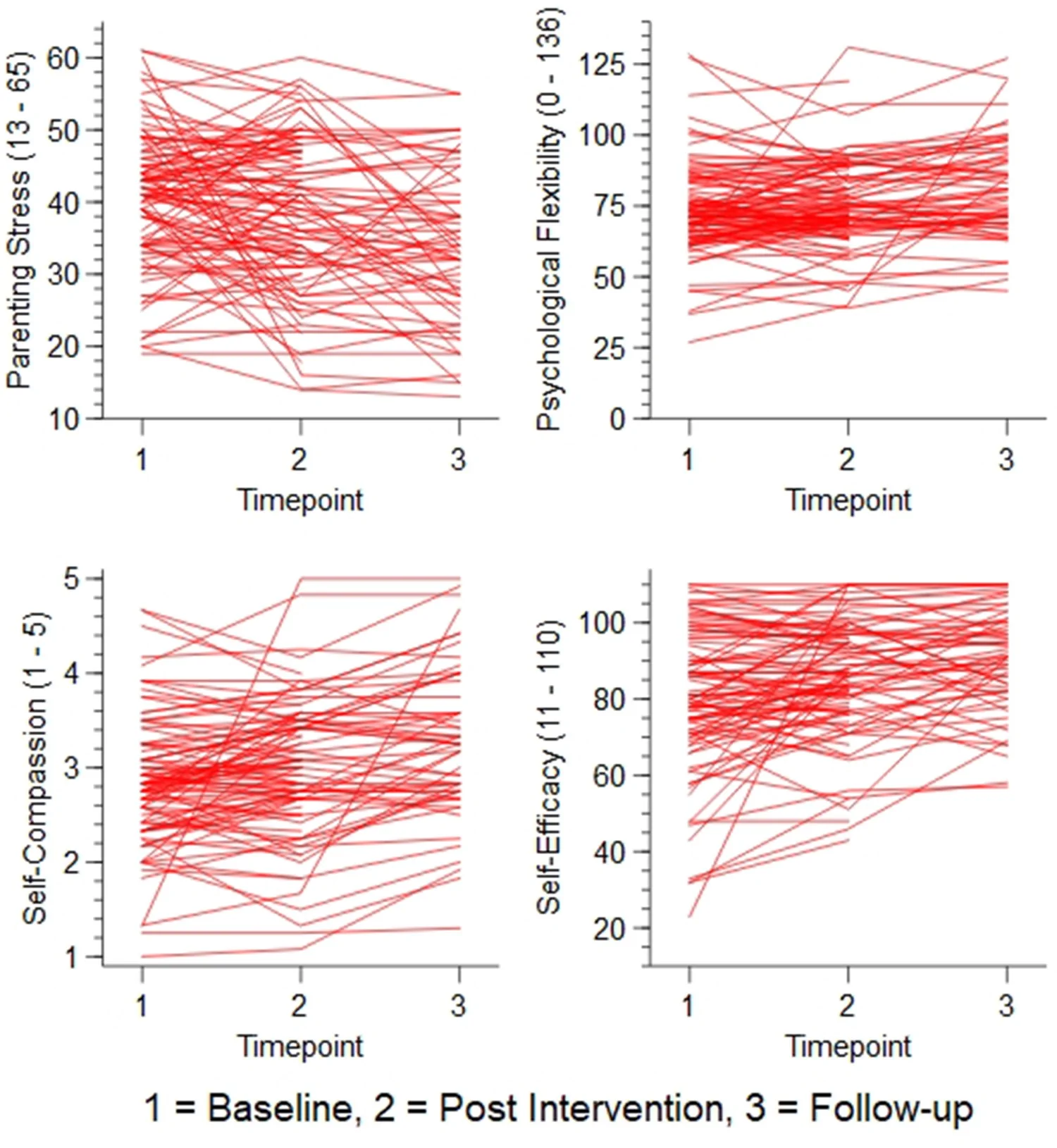
Participants’ scores at each timepoint.

The graphs in Figure 2 show that, although effects are not universal, more participants for whom we have matched data on at least two time points improved than did not improve.

### User engagement

Parents/carers reported which sections of the toolkit they accessed. Of the 104 respondents at post-intervention, 89 (85.6%) had engaged with “Introduction (why support parents?)”. Other sections accessed included “What are difficult thoughts and feelings?” (*n *= 79, 76%), “Helper skills” *(n *= 81, 77.9%), “Being present” (*n *= 73, 70.2%), “Knowing your values” (*n *= 70, 67.3%), and “Talking about your child’s condition or injury” (*n *= 73, 70.2%). At follow-up, the percentage of parents/carers who had engaged with each of these sections was 74%, 70.1%, 67.5%, 79.2%, 66.2%, and 79.2%, respectively. Parents/carers were also asked whether they had tried any of the exercises in the toolkit. At post-intervention, 67 (64.4%) reported that they had tried some exercises, and at follow-up, 56 (72.7%) had done so. At post-intervention and follow-up, parents/carers responded to a question about what format they would access the toolkit in. At post-intervention, 93 (89.4%) indicated that they would access it as an e-book, aligning with the current format, and 32 (30.8%) reported they would access it as a physical booklet. At follow-up, these numbers were similar, with 67 (87%) reporting they would access the toolkit as an e-book and 22 (28.6%) indicating they would access it as a physical booklet.

### Open-ended questions

Three main categories were generated from content analysis of open-ended survey responses. In “Making a difference,” parents/carers expressed gratitude for the toolkit, reporting increased knowledge, confidence, and understanding. This main category included a subcategory, “Dyadic benefits,” which reflected how engaging with the toolkit improved both their own well-being and that of their child. As a result of managing their own challenges, they reported feeling that they could better support their child. The second main category, “Parent worries persist,” described that despite the benefits of the toolkit, many parents/carers still held concerns and worries about their child’s future and the responsibility of wanting to support their child in the best way possible. Finally, the third main category, “Challenges engaging others,” described difficulties sharing the intervention, such as the child being too young or a partner being less engaged. See Table 4 for categories and example quotes.

**Table 4:** Summary of content analysis categories with example quotes.

Category	Frequency of category codes	Subcategory	Frequency of subcategory codes	Description	Example quotes
“Making a difference”	30	Dyadic benefits—the concept that the toolkit has helped parents to foster stronger relationships with their children and improved their communication and connection. Quote: “…this toolkit is helping so I can become more confident myself as well as being the best role model to my son”	7	Parents reported that the toolkit helped them to feel more knowledgeable about their child’s condition, as well as more confident about advocating for them and making informed decisions.	“Thank you for this … has made a real difference to me” “…think the parenting tool kit has been amazing at helping me feel equipped with helping me manage the challenges we face…”
Parent worries persist	9	N/A		Parents highlighted the uncertainty for the future, feeling conflicted over how best to support their children, and the responsibility they feel for their children’s experiences as key challenges and worries.	“Feelings of being an inadequate parent because I feel I can’t help him to feel better.” “…and I worry very much about his future and the decisions we are faced with as parents.”
Challenges engaging others	8	N/A		Some noted the difficulties that can exist for engaging others in the content, for example, partners/other caregivers or very young children.	“My husband seems to be unable to see past the unfairness of the situation and would benefit more from your guide than me—however he’s resistant to anything that doesn’t fix the problem… ‘it’s all just words”

## Discussion

This study investigated the acceptability and potential benefit of The Visible Difference Parenting Toolkit in reducing stress and improving psychosocial well-being for parents and carers of children with a visible difference of any kind. Significant decreases in parent-reported depression and anxiety and increases in parenting self-efficacy were found between baseline and post-intervention and baseline and follow-up. A significant decrease in parenting stress and increases in self-compassion and psychological flexibility (openness to experience and valued action) were found between baseline and follow-up.

An ACT approach emphasizes the importance of practice and time, which may explain why outcomes in this study improved over time. The intervening time between post-intervention and follow-up presented an opportunity to apply, consolidate, and derive benefit from the intervention, which may explain the significant positive changes in parenting stress, psychological flexibility, and self-compassion at the 2-month follow-up but not post-intervention.

Significant changes in psychosocial outcomes suggest the toolkit was beneficial in supporting parent/carer well-being. Open-ended data reinforced that parents/carers felt more equipped to manage their own well-being and support their child. This aligns with evaluations of ACT interventions for parents/carers of children with long-term conditions. For example, a systematic review found that ACT-based interventions were effective in increasing psychological flexibility and reducing distress in parents/carers of children with chronic conditions (Jin et al., 2021). Another evaluation showed improvements in parent self-compassion and child well-being (Ghorbanikhah et al., 2023). While ACT-based interventions may benefit both parents/carers and children, child outcomes were not assessed in this study, so further research is needed to explore systemic impact in families of children with a visible difference.

Despite demonstrating that the toolkit is associated with improvements in psychosocial outcomes, some parents/carers remained concerned about future challenges. This reflects the ongoing nature of psychosocial difficulties experienced by parents/carers of children with visible differences, as noted in existing literature (e.g., Oliver et al., 2020; Thornton et al., 2021; Zelihić et al., 2021). Previous research highlights that while early years can be challenging (Costa et al., 2021; Heath et al., 2018; Thornton et al., 2024), adolescence and young adulthood bring evolving parenting difficulties (Stock et al., 2015). Open-ended responses indicated that some parents/carers of younger children struggled to engage with certain sections of the toolkit, such as parent–child communication, due to developmental stage. These findings highlight the need for developmentally appropriate support across the child’s lifespan.

The present study has demonstrated non-trivial statistically significant improvements at two-month follow-up, with effect sizes in the range of Cohen’s *d* = 0.31–0.49. These findings are further strengthened by strong correlations between changes in psychological flexibility from baseline to follow-up and corresponding changes in anxiety, depression, parenting stress, parenting self-efficacy, and self-compassion. These large correlations are consistent with proposed mechanisms of change and add credibility to findings. Nevertheless, the limitations of the single-arm design warrant caution in interpreting these results.

As a self-guided intervention and as indicated by user engagement data, participants accessed different sections based on time and needs. Some sections (Introduction, Helper skills, Difficult thoughts and feelings, Talking about your child’s condition or injury) were more frequently used. It is possible that these were the sections that participants thought were most relevant to them. Most participants had also engaged in some exercises. This likely contributed to the significant impact of the toolkit on parent well-being, as parents/carers seemed to be invested and engaged throughout the intervention.

It is possible that parents/carers could benefit more from engaging with the toolkit over a longer period. However, intervention development and implementation theory highlights the complexity of “dose response” in that the ‘dose’ (i.e., the level of engagement with the intervention) that confers benefits varies between individuals. Outcomes may depend more on the quality of delivery and individual resonance rather than cumulative exposure and whether there is an immediate or delayed need for support. It would be premature to conclude, on the basis of the current study, that greater engagement with the toolkit necessarily improves outcomes, and further research is needed to examine this in more detail. In the meantime, we tentatively suggest that future users be encouraged to engage with and utilize all aspects of the intervention.

Regarding delivery mode, most respondents (*n *= 93; 89% post-intervention) indicated that they preferred the e-book format. This supports findings that digital ACT-based interventions are acceptable to the visible difference population (Zucchelli et al., 2022). Furthermore, a systematic review of interventions for adults with a range of chronic health conditions found that online self-guided ACT interventions can be an effective transdiagnostic approach for improving psychological well-being, quality of life, and psychological flexibility (Klimczak et al., 2023). Despite this, some research suggests that an online ACT-based intervention delivered with facilitation from a professional (e.g., online meetings with a psychologist) can lead to more significant improvements in psychosocial well-being (Lappalainen et al., 2021). However, access to specialist psychological support for those affected by visible difference is limited (Harcourt et al., 2018). Evidence also suggests self-help parenting interventions can be a cost-effective way to increase access to psychosocial support (Daley & O’Brien, 2013). Consequently, an online accessible format for The Visible Difference Parenting Toolkit is potentially effective in supporting parent/carer psychosocial well-being, acceptable to the target group, and allows the support to be widely accessible.

### Implications

The present research demonstrates that ACT is an appropriate therapeutic approach for this population and indicates which psychosocial constructs may be effective targets for intervention (e.g., psychological flexibility and self-compassion). The Visible Difference Parenting Toolkit is a free and accessible e-book, which provides healthcare professionals and support organizations with an evidence-based online resource to signpost parents/carers to, addressing an important gap in unmet support needs for parents/carers (Thornton et al., 2026). Therefore, this is a novel and important contribution to the psychosocial support available for parents and carers of children with a visible difference.

The Visible Difference Parenting Toolkit could be incorporated into existing services offering psychosocial support, including healthcare providers and support organizations. Parents/carers could initially be helped to formulate a plan for how to use the toolkit, including simple time management such as how to make a plan and set aside time, set goals, and develop “if then” rules, as with other behavior change programmes. This would aim to facilitate their use of The Visible Difference Parenting Toolkit, possibly alongside other self-management tools. Those who use the toolkit as intended but still report difficulties and distress would need formal assessment with psychological services and a higher-order intervention, as per stepped models of care.

### Limitations

While this study presents a novel intervention for supporting parents/carers of children with a visible difference, several limitations must be acknowledged. Firstly, the sample was self-selecting and may not represent all parents/carers who might require support. Most participants (*n *= 126, 72%) were women and the child’s mother, a common trend in parenting research within the visible difference field (e.g., Heath et al., 2018; Hlongwa & Rispel, 2018; Razera et al., 2017) and in parenting research more broadly (e.g., Jin et al., 2021; Lappalainen et al., 2021). Due to this gender imbalance, it is unclear whether the toolkit is potentially beneficial for carers of all genders or roles. Research suggests men may accept practical help but often downplay the emotional impact of managing health challenges (Flurey et al., 2023), so future research should consider whether an adapted approach to providing support to fathers may be more accessible and engaging.

The majority of participants also identified as White (*n *= 123, 70%). The limited research from non-Western cultures has demonstrated variations in the experiences of parents of children with a visible difference (e.g., differences in causal beliefs; Olasoji et al., 2007). Individuals from minoritized racialized groups may have differing support needs not captured by the existing literature. As such, we may not be able to generalize the findings of the present study to parents and carers from other cultural backgrounds.

Additionally, attrition in the study was high. Although missing values analysis indicated that there were no significant differences in demographic characteristics or baseline well-being measures between those who were retained and those who dropped out, it remains possible that other differences existed that contributed to certain participants dropping out. As such, the results may not be generalizable to all parents and carers of children with visible differences. It is possible that participants who felt they had benefited from the intervention were more likely to complete follow-up measures, and systematic regression to the mean may also have contributed, as participants with more extreme baseline scores may be expected to show some natural progression to the population mean at later time points, irrespective of intervention.

Given the positive comments we received from participants, it is possible that some might have continued to use the toolkit beyond the study period, but we do not have data on this. Similarly, we relied on self-reports and do not have objective data on when and how participants engaged with the toolkit.

Although the toolkit has been assessed as being at a reading age of 12 years old or 8th grade, participants’ reading ability was not assessed, and it is possible that parents/carers who have lower literacy levels and might benefit from the toolkit did not take part. The toolkit is also currently only available in English, which may have excluded some parents/carers from taking part. Future research and dissemination should consider translating The Visible Difference Parenting Toolkit into other languages and formats (e.g., physical booklets, audio versions) to increase accessibility.

Informed by feedback from the parent/carer advisory group, the study did not include a control or comparison group. Other intervention studies within the field of visible difference have utilized control groups (e.g., Van Dalen et al., 2021); however, given the lack of support accessible to parents/carers, the intervention was provided to all participants. As a comparison is not available within the present study, it may not be possible to conclude whether the changes observed between baseline, post-intervention, and follow-up are due entirely to exposure to the intervention or due to other factors.

## Conclusion

This preliminary mixed-methods evaluation aimed to assess the potential benefit of The Visible Difference Parenting Toolkit, a self-guided ACT-based intervention aimed at improving parental stress and psychosocial well-being for parents and carers of children with visible differences. It was found that parent-reported depression and anxiety significantly decreased, and parenting self-efficacy significantly increased between baseline and post-intervention. Parent-reported depression, anxiety, and stress also significantly decreased, and self-compassion, self-efficacy, and psychological flexibility significantly increased between baseline and follow-up. Parents/carers reported in open-ended questions that The Visible Difference Parenting Toolkit helped them to feel more knowledgeable and more confident in supporting their child. These findings suggest that this novel self-guided ACT-based intervention is acceptable and potentially beneficial for supporting psychosocial well-being for this population. Future research could explore the effectiveness for parents and carers of a broader range of genders and ethnicities and consider providing the toolkit in other languages and formats to increase reach and accessibility. The Visible Difference Parenting Toolkit can now be accessed, free of charge, at www.VisibleDifferenceSupportHub.com.

## Supplementary Material

jsag043_Supplementary_Data

## Data Availability

Due to ethical restrictions, data cannot be shared. Data includes outcomes and demographic information provided by with parents of children with visible differences (some of which are rare conditions) and sharing the data may result in identification of these individuals.
